# Supplementary material for: Inference and effects of barcode multiplets in droplet-based single-cell assays
Source: Nat Commun. 2020 Feb 13;11:866. doi: 10.1038/s41467-020-14667-5 (PMC7018801; doi:10.1038/s41467-020-14667-5)
Supplement: Supplementary file 1 — Supplementary Information [file 41467_2020_14667_MOESM1_ESM.pdf]

Supplementary information for

Inference and effects of barcode multiplets in droplet-based single-cell assays

Caleb Lareau, Sai Ma, Fabiana Duarte, Jason Buenrostro

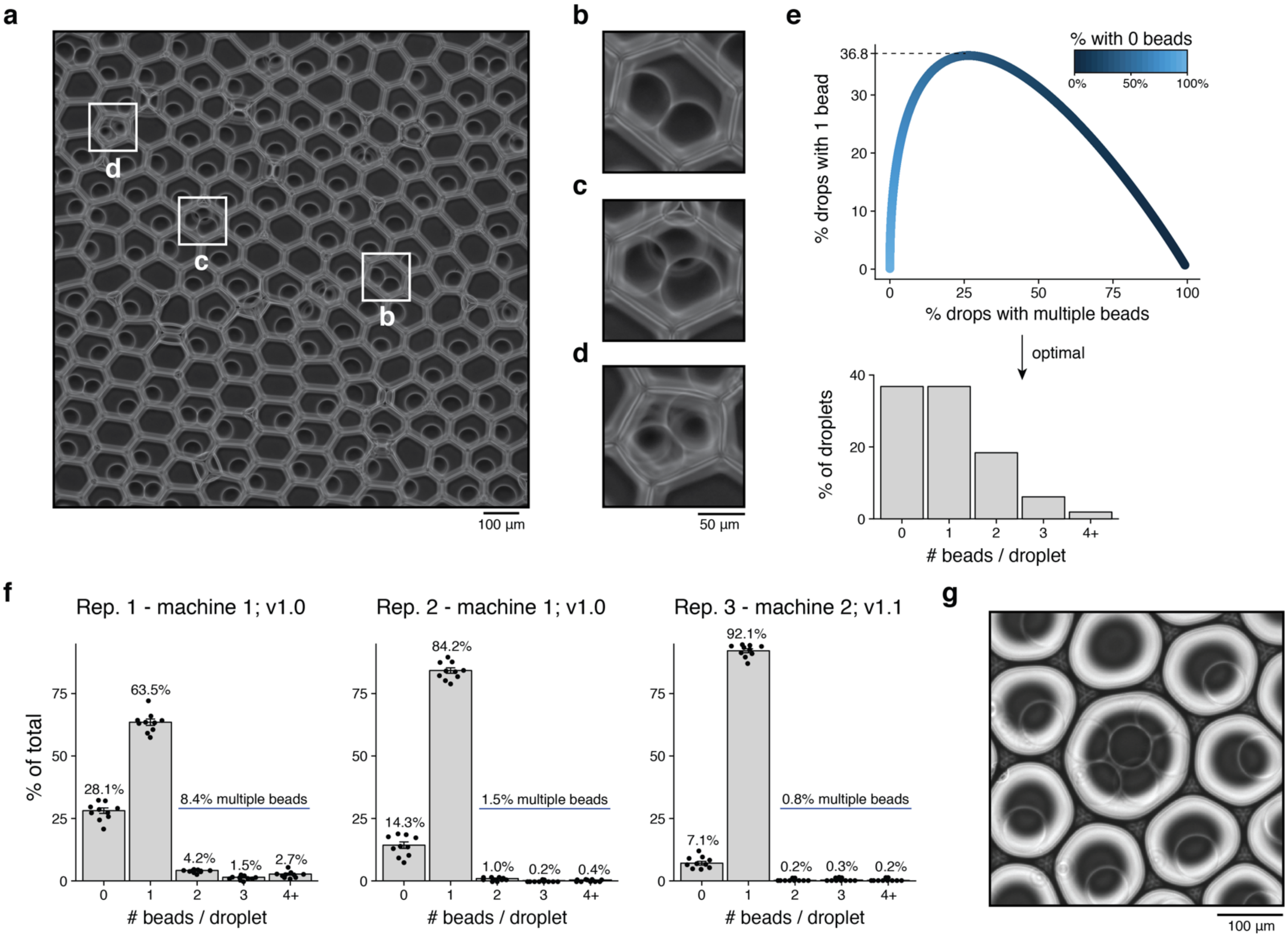

Supplementary Figure 1 - Supporting information for Figure 1. (a) Alternative field of view. Boxes highlight individual droplets shown in subsequent panels. The image is representative of a total of 30 fields of view taken from 3 independent experiments. **(b-d)** Examples of 2, 3, and 4+ beads per droplet, respectively. **(e)** Theoretical support for optimal bead loading under Poisson distribution assumptions. The dotted line (top) represents the theoretical maximum for 1 bead loaded into droplets, and the full distribution at this point is shown in the bar graph. **(f)** Quantification of beads per droplet for each replicate. Above each panel, the machine and the version of the chip used for the training kit is indicated. Error bars represent standard error of mean over n=10 independent fields of view for each of the three experimental replicates (n=30 total). **(g)** Example of presumed merged droplet containing multiple (6) beads. The selected droplet was one of ~10 droplets that was likely the consequence of merging taken from a total of 30 fields of view taken from 3 independent experiments.

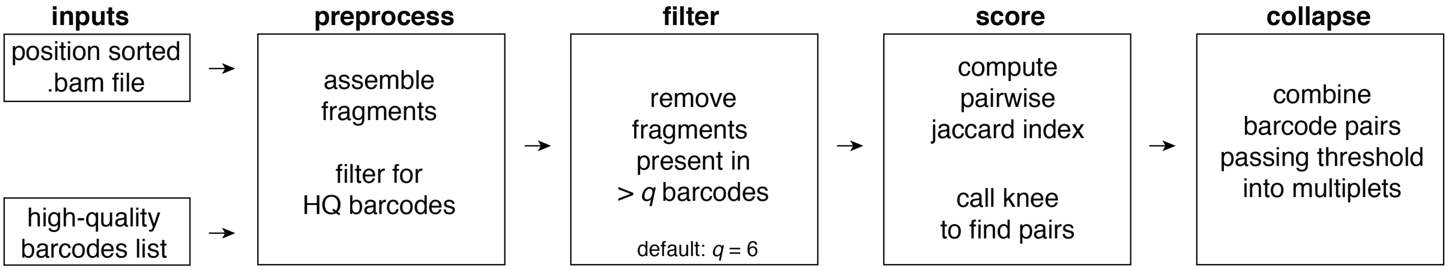

Supplementary Figure 2 - Supporting information for Figure 2. An overview of the inputs and computational workflow for the application of bap to 10x scATAC-seq data.

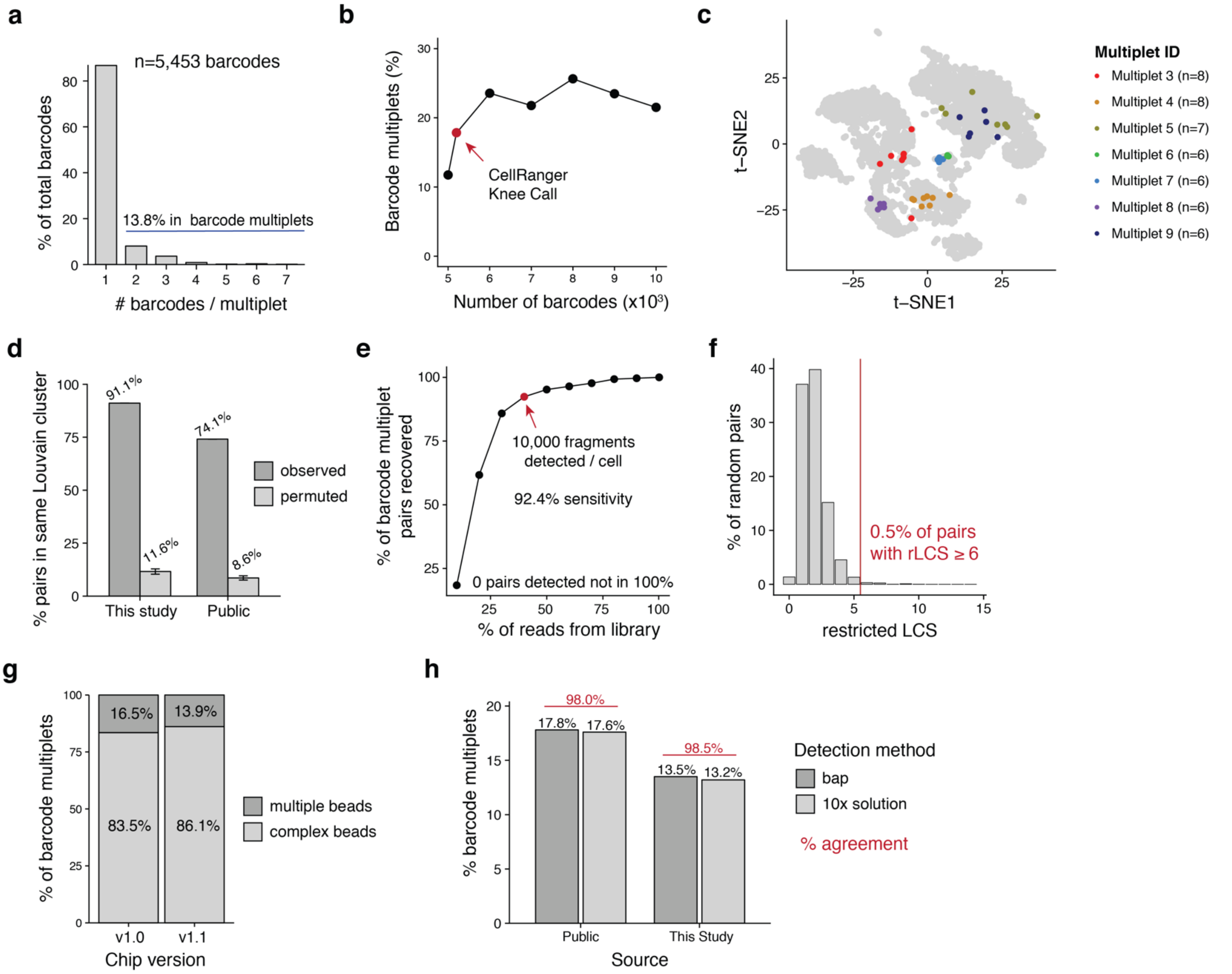

Supplementary Figure 3 - Supporting information for Figure 3. (a) Quantification of barcodes affected by barcode multiplets for the PBMC dataset generated with this work (“This Study”). **(b)** Percentage of barcode multiplets identified for different numbers of input barcodes (see **Methods**). **(c)** Visualization of seven additional barcode multiplets from the Public dataset. **(d)** Proportion of bead pairs occurring in the same chromatin accessibility-defined Louvain cluster compared to a permuted background. Error bars represent standard error of mean over n=100 independent permutations per each dataset (two independent experimental replicates). **(e)** Downsampling analysis of the dataset generated in this work (“This Study”). Barcode multiplets were examined at downsampled intervals from 10%-90% by units of 10%. The highlighted sample represents 40% downsampling and corresponds to a median 10,000 fragments detected per barcode. At all downsampled thresholds, we detected 0 pairs that were not present in the 100% sample. **(f)** Distribution of the restricted longest common subsequence (rLCS) for 1,000,000 randomly sampled barcode pairs in the 10x barcode universe. A threshold at 6 is drawn for use in other analyses. **(g)** Breakdown of types of barcode multiplets from the Next-gem comparison data. **(h)** Comparison of methods to detect barcode multiplets. The rates of barcode multiplets detected by each solution is shown in black. The % agreement between the two methods (per barcode) is shown in red.

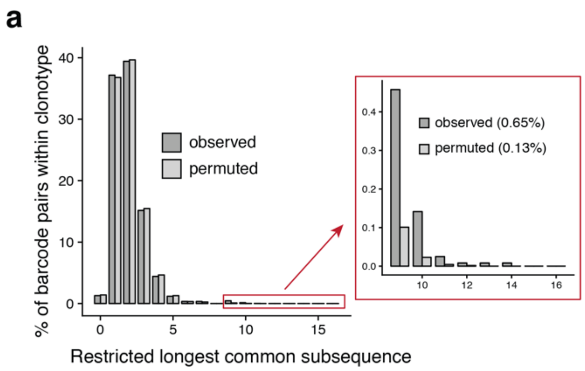

Supplementary Figure 4 - Supporting information for Figure 4. (a) Observed and permuted (within clonotype) restricted longest common subsequence for the BCR clone dataset. The inset shows a zoom for rLCS ≥9 and the percent of barcodes depicted in the panel.

Supplementary Table 1 - Quantifications of bead abundances in droplets across 30 fields of view.

Experiment 1 - v1 beads										
# beads	**FOV1**	**FOV2**	**FOV3**	**FOV4**	**FOV5**	**FOV6**	**FOV7**	**FOV8**	**FOV9**	**FOV10**
**0**	55	54	49	52	57	44	32	41	45	55
**1**	112	117	115	114	101	109	110	110	104	101
**2**	9	8	8	8	5	8	6	6	7	7
**3**	5	3	1	3	2	4	0	3	2	2
**≥4**	3	2	5	3	10	4	4	6	5	5

Experiment 2 - v1 beads										
# beads	**FOV1**	**FOV2**	**FOV3**	**FOV4**	**FOV5**	**FOV6**	**FOV7**	**FOV8**	**FOV9**	**FOV10**
**0**	25	18	20	19	23	12	21	9	11	22
**1**	107	115	121	113	108	100	96	106	102	91
**2**	1	1	2	1	0	1	0	2	2	2
**3**	0	1	0	0	0	0	0	1	0	0
**≥4**	0	1	1	1	0	1	0	0	1	0

Experiment 3 - v1.1 beads										
# beads	**FOV1**	**FOV2**	**FOV3**	**FOV4**	**FOV5**	**FOV6**	**FOV7**	**FOV8**	**FOV9**	**FOV10**
**0**	9	4	7	6	4	8	6	8	6	5
**1**	66	80	72	83	84	76	89	89	86	91
**2**	0	0	0	0	0	0	0	1	0	1
**3**	1	0	0	0	1	0	0	0	1	0
**≥4**	0	1	0	0	0	1	0	0	0	0

Supplementary Table 2 - Highlighted barcode multiplets in Fig. 3d and S3c.

Barcode	bap_id	n_beads	Cluster	TSNE.1	TSNE.2	MultipletID
TTTGCGCCAAACCCTA-1	BC3848_N09	9	7	-16.519768	-26.145098	Multiplet1
TTTGCGCCAAAGACGC-1	BC3848_N09	9	7	-16.469458	-25.706735	Multiplet1
TTTGCGCCAAGACTTC-1	BC3848_N09	9	7	-12.705563	-25.161819	Multiplet1
TTTGCGCCAAGTCTGT-1	BC3848_N09	9	7	-16.531485	-23.153162	Multiplet1
TTTGCGCCACAGTAGG-1	BC3848_N09	9	7	-16.256563	-25.87321	Multiplet1
TTTGCGCCACGAACGA-1	BC3848_N09	9	7	-13.997486	-23.582679	Multiplet1
TTTGCGCCAGAACAGC-1	BC3848_N09	9	7	-16.755185	-26.088484	Multiplet1
TTTGCGCCAGTTCGGC-1	BC3848_N09	9	7	-16.294488	-25.997215	Multiplet1
TTTGCGCCATCGGCCA-1	BC3848_N09	9	7	-16.763635	-26.782414	Multiplet1
AACCGATAGAGTGGAA-1	BC2220_N09	9	2	3.3160166	29.028777	Multiplet2
AGTCCGGAGAGTGGAA-1	BC2220_N09	9	2	-2.064109	18.927084	Multiplet2
CGTAAACAGAGTGGAA-1	BC2220_N09	9	2	5.3009276	20.871638	Multiplet2
CTGTATTAGAGTGGAA-1	BC2220_N09	9	2	-0.3873512	27.8031	Multiplet2
GCTCACTAGAGTGGAA-1	BC2220_N09	9	2	-5.0370618	21.856891	Multiplet2
GGAACTTAGAGTGGAA-1	BC2220_N09	9	2	-4.8989146	21.055038	Multiplet2
TAATCGGAGAGTGGAA-1	BC2220_N09	9	2	-3.2995225	22.321848	Multiplet2
TCCAGAAAGAGTGGAA-1	BC2220_N09	9	2	4.5209052	14.076471	Multiplet2
TTGTTCAAGAGTGGAA-1	BC2220_N09	9	2	3.567621	18.975025	Multiplet2
AAAGGATAGTCAACTC-1	BC2312_N08	8	10	-8.1173794	-5.214104	Multiplet3
ATGTCGAAGTCAACTC-1	BC2312_N08	8	10	-12.296727	-4.608763	Multiplet3
CATAACGAGTCAACTC-1	BC2312_N08	8	10	-8.1355065	-4.004572	Multiplet3
GGAATCTAGTCAACTC-1	BC2312_N08	8	10	-5.4273919	5.472192	Multiplet3
GGCACGTAGTCAACTC-1	BC2312_N08	8	10	-5.4896231	-28.22882	Multiplet3
GTCACAAAGTCAACTC-1	BC2312_N08	8	10	-16.037159	-7.647751	Multiplet3
GTCACCTAGTCAACTC-1	BC2312_N08	8	10	-8.6424666	-6.181868	Multiplet3
TATCGAGAGTCAACTC-1	BC2312_N08	8	10	-8.033321	-3.814572	Multiplet3
AACCGATAGCGTTGCC-1	BC0373_N08	8	3	-1.3758117	-20.5985	Multiplet4
AGTCCGGAGCGTTGCC-1	BC0373_N08	8	3	-5.1764372	-20.9297	Multiplet4
CAGGATTAGCGTTGCC-1	BC0373_N08	8	3	1.6606752	-20.4978	Multiplet4
GCATTGAAGCGTTGCC-1	BC0373_N08	8	3	7.3648887	-19.4181	Multiplet4
GGAACTTAGCGTTGCC-1	BC0373_N08	8	3	-2.0064488	-23.7749	Multiplet4
TAATCGGAGCGTTGCC-1	BC0373_N08	8	3	-4.6265402	-21.3103	Multiplet4

TAGACTGAGCGTTGCC-1	BC0373_N08	8	3	0.6822699	-23.4100	Multiplet4
TTGTTCAAGCGTTGCC-1	BC0373_N08	8	3	-0.2318434	-19.9549	Multiplet4
AACCGATAGTTACCAC-1	BC4076_N07	7	1	26.0246446	7.310766	Multiplet5
CGTAAACAGTTACCAC-1	BC4076_N07	7	4	14.9570596	19.602828	Multiplet5
CTGTATTAGTTACCAC-1	BC4076_N07	7	4	36.7388061	10.530711	Multiplet5
GCTCACTAGTTACCAC-1	BC4076_N07	7	5	26.6666906	6.358799	Multiplet5
GGAACTTAGTTACCAC-1	BC4076_N07	7	1	6.0165494	11.406972	Multiplet5
TAATCGGAGTTACCAC-1	BC4076_N07	7	1	23.3612255	7.21295	Multiplet5
TCCAGAAAGTTACCAC-1	BC4076_N07	7	2	4.659057	13.466048	Multiplet5
CACCTGTTCCTCCTGA-1	BC4341_N06	6	1	6.5278998	-4.663518	Multiplet6
CTCAGAACAGAACAGC-1	BC4341_N06	6	13	6.736109	-4.698881	Multiplet6
GGTCATACAAACCTAC-1	BC4341_N06	6	1	6.562749	-4.547282	Multiplet6
TACTCGCAGTCCCTCT-1	BC4341_N06	6	2	7.0396085	-4.784727	Multiplet6
TAGACTGGTGTGAGGT-1	BC4341_N06	6	1	6.7331135	-4.603496	Multiplet6
TATTGCTCACGGCCAT-1	BC4341_N06	6	1	6.6004841	-4.330638	Multiplet6
AACGTACTCTGGCTAA-1	BC2839_N06	6	13	3.9715859	-5.889223	Multiplet7
ACAGCGCGTAATGCCT-1	BC2839_N06	6	8	3.8632771	-5.211903	Multiplet7
ACAGCGCGTCAGACGA-1	BC2839_N06	6	13	4.1422006	-6.240332	Multiplet7
ACAGCGCGTCGAGGAT-1	BC2839_N06	6	13	3.8024788	-7.004544	Multiplet7
ACAGCGCGTGAGGTCA-1	BC2839_N06	6	6	3.3131449	-6.041366	Multiplet7
ACAGCGCGTTTAGAAG-1	BC2839_N06	6	13	5.130589	-5.865072	Multiplet7
TTAGGTGGTAATGCCT-1	BC0809_N06	6	7	-15.9006	-24.1150	Multiplet8
TTAGGTGGTCAGACGA-1	BC0809_N06	6	7	-16.719811	-24.9081	Multiplet8
TTAGGTGGTCGAGGAT-1	BC0809_N06	6	7	-14.775208	-22.7837	Multiplet8
TTAGGTGGTGAGGTCA-1	BC0809_N06	6	7	-14.664229	-24.0957	Multiplet8
TTAGGTGGTTGGAGGT-1	BC0809_N06	6	7	-19.179605	-20.7603	Multiplet8
TTAGGTGGTTTAGAAG-1	BC0809_N06	6	7	-15.986716	-22.8054	Multiplet8
GTCACTCTCACCCTTG-1	BC4330_N06	6	1	14.2027744	3.917249	Multiplet9
GTCACTCTCAGGATCT-1	BC4330_N06	6	1	19.0922335	12.623059	Multiplet9
GTCACTCTCCAACAAC-1	BC4330_N06	6	1	19.6237027	8.267061	Multiplet9
GTCACTCTCCCGTAGG-1	BC4330_N06	6	1	23.4451766	2.518076	Multiplet9
GTCACTCTCCTTACGC-1	BC4330_N06	6	1	10.7239372	10.051789	Multiplet9
GTCACTCTCGATAGGG-1	BC4330_N06	6	1	13.7965715	2.689037	Multiplet9

Supplementary Table 3 - Supporting information for simulation parameterization.

Table of multiplet values per dataset			
Barcode multiplicity*	**Public**	**This Study**	**Simulation**
1	0.925	0.87	0.93
2	0.045	0.08	0.05
3	0.02	0.035	0.01
4	0.005	0.01	0.005
≥5	0.005	0.005	0.005
Estimated barcode multiplet rate	17.6%	13.2%	15.8%

*The barcode multiplicity rate corresponds to the m_i parameter used in the simulation.
It can be interpreted as the number of barcodes in a particular droplet
The multiplet rate is the percent of barcodes affected by the multiplet artifact
