# Supplementary material for: Inference and effects of barcode multiplets in droplet-based single-cell assays
Source: Nat Commun. 2020 Feb 13;11:866. doi: 10.1038/s41467-020-14667-5 (PMC7018801; doi:10.1038/s41467-020-14667-5)
Supplement: Supplementary file 2 — Reporting Summary [file 41467_2020_14667_MOESM2_ESM.pdf]

nature research

Corresponding author(s): Lareau, Buenrostro

Last updated by author(s): 1/19/2020

Reporting Summary

Nature Research wishes to improve the reproducibility of the work that we publish. This form provides structure for consistency and transparency in reporting. For further information on Nature Research policies, see Authors & Referees and the Editorial Policy Checklist .

Statistics

For all statistical analyses, confirm that the following items are present in the figure legend, table legend, main text, or Methods section.

n/a	Confirmed	
☐	☒	The exact sample size (*n*) for each experimental group/condition, given as a discrete number and unit of measurement
☐	☒	A statement on whether measurements were taken from distinct samples or whether the same sample was measured repeatedly
☐	☒	The statistical test(s) used AND whether they are one- or two-sided<br>*Only common tests should be described solely by name; describe more complex techniques in the Methods section.*
☒	☐	A description of all covariates tested
☐	☒	A description of any assumptions or corrections, such as tests of normality and adjustment for multiple comparisons
☐	☒	A full description of the statistical parameters including central tendency (e.g. means) or other basic estimates (e.g. regression coefficient) AND variation (e.g. standard deviation) or associated estimates of uncertainty (e.g. confidence intervals)
☐	☒	For null hypothesis testing, the test statistic (e.g. *F*, *t*, *r*) with confidence intervals, effect sizes, degrees of freedom and *P* value noted<br>*Give P values as exact values whenever suitable.*
☒	☐	For Bayesian analysis, information on the choice of priors and Markov chain Monte Carlo settings
☒	☐	For hierarchical and complex designs, identification of the appropriate level for tests and full reporting of outcomes
☒	☐	Estimates of effect sizes (e.g. Cohen's *d*, Pearson's *r*), indicating how they were calculated

Our web collection on statistics for biologists contains articles on many of the points above.

Software and code

Policy information about availability of computer code

Data collection: Complete code and documentation for the software suite developed in this study (bap - bead-based ATAC-seq processing tool) is available on GitHub under the following weblink: https://github.com/caleblareau/bap. Version 0.5.9+ facilitates analysis with the 10X scATAC-seq data. To process the scATAC-seq data from raw .fastq files, we used CellRanger-ATAC version 1.0.0.

Data analysis: Scripts corresponding to the analyses contained in this paper are further provided at: https://github.com/caleblareau/barcode-multiplets.

For manuscripts utilizing custom algorithms or software that are central to the research but not yet described in published literature, software must be made available to editors/reviewers. We strongly encourage code deposition in a community repository (e.g. GitHub). See the Nature Research guidelines for submitting code & software for further information.

Data

Policy information about availability of data

All manuscripts must include a data availability statement. This statement should provide the following information, where applicable:
- Accession codes, unique identifiers, or web links for publicly available datasets
- A list of figures that have associated raw data
- A description of any restrictions on data availability

Software associated with the barcode multiplet identification and merging algorithm is available at https://github.com/caleblareau/bap. Code and data to reproduce the main findings of this study are available at https://github.com/caleblareau/barcode-multiplets. The public 10x scATAC-seq datasets are available for download at https://support.10xgenomics.com/single-cell-atac/datasets and the public NSCLC clontypes at https://www.10xgenomics.com/solutions/vdj/. Sequencing data generated as part of this work is available at the Gene Expression Omnibus under accession GSE143197.

Field-specific reporting

Please select the one below that is the best fit for your research. If you are not sure, read the appropriate sections before making your selection.

☒ Life sciences ☐ Behavioural & social sciences ☐ Ecological, evolutionary & environmental sciences

For a reference copy of the document with all sections, see nature.com/documents/nr-reporting-summary-flat.pdf

Life sciences study design

All studies must disclose on these points even when the disclosure is negative.

Sample size	No sample size calculations were performed. We highlight two different single cell datasets (each containing > 5,000 cells) as they were generated at different institutions to ensure the confounding effect was not due to a particular machine. We suggest that this provides a sufficient sample size of cells as 5,000 is generally a recommended output by the manufacturers of the technology.
Data exclusions	No data were excluded in these analyses.
Replication	We were able to successfully replicate the barcode multiplet effect in data generated locally ("This Study") after first observing the phenomenon in public data. The replication experiment was performed once but yielded > 5,000 "cells".
Randomization	There were no variables to randomize in this study. Associations between barcode multiplets and other variables (e.g. cluster) were performed by independent software than used to identify multiplets.
Blinding	Blinding is not relevant to our study, as our statistical tools are not dependent on blinding. Investigators could not be blinded during data collection or analysis as there was no intervention. Further, analyses were performed in an exploratory manner where blinding is not possible.

Reporting for specific materials, systems and methods

We require information from authors about some types of materials, experimental systems and methods used in many studies. Here, indicate whether each material, system or method listed is relevant to your study. If you are not sure if a list item applies to your research, read the appropriate section before selecting a response.

Materials & experimental systems

n/a	Involved in the study
☒	☐ Antibodies
☒	☐ Eukaryotic cell lines
☒	☐ Palaeontology
☒	☐ Animals and other organisms
☒	☐ Human research participants
☒	☐ Clinical data

Methods

n/a	Involved in the study
☒	☐ ChIP-seq
☒	☐ Flow cytometry
☒	☐ MRI-based neuroimaging
